# Reproductive Biology and Distribution of the Blue Shark (*Prionace glauca*) in the Western Indian Ocean

**DOI:** 10.3390/biology12081128

**Published:** 2023-08-14

**Authors:** Jizhang Zhu, Zhe Geng, Jiangfeng Zhu, Kindong Richard

**Affiliations:** 1 College of Marine Sciences, Shanghai Ocean University, Shanghai 201306, China; zjz2373@gmail.com (J.Z.); zgeng@shou.edu.cn (Z.G.); 2 Key Laboratory of Sustainable Exploitation of Oceanic Fisheries Resources, Ministry of Education, Shanghai 201306, China; 3 Key Laboratory of Oceanic Fisheries Exploration, Ministry of Agriculture and Rural Affairs, Shanghai 201306, China; 4 National Engineering Research Centre for Oceanic Fisheries, Shanghai Ocean University, Shanghai 201306, China

**Keywords:** blue shark, Indian Ocean, reproductive biology, spatiotemporal distribution, fishery observer program, pelagic fisheries, habitat

## Abstract

**Simple Summary:**

Blue shark populations are currently declining, caused mainly by the impact of overfishing from industrial fishing vessels in the open oceans. However, there is a paucity of comprehensive biological and habitat data concerning the reproductive characteristics of blue shark species in the Indian Ocean. In response to the call made by the Indian Ocean Tuna Commission (IOTC) to provide valuable parameters for the conservation of blue shark resources, this study collected observer data from the Indian Ocean longline fishery targeting tuna between 2010 and 2020. Through rigorous statistical analysis, it was determined that the estimated length at sexual maturity for male blue sharks is 161.4 cm and 179.3 cm for females. Moreover, the average litter size was found to be 33.7 pups. The study suggests the existence of a possible seasonal migratory pattern for pregnant blue sharks, with the first and fourth quarters of the year being potential mating grounds in proximity to the equatorial region of the Indian Ocean. Additionally, the temperate marine areas of the Indian Ocean were identified as crucial habitats for parturition and juvenile development of blue shark species. Consequently, it is strongly recommended to implement more scientifically informed and ecologically sustainable operational strategies in these designated areas.

**Abstract:**

Due to the limited biological research on the blue shark in the Indian Ocean, such as the lack of a clear understanding of its reproductive biology and distribution, our study analyzed and evaluated the fork length distribution, sexual maturity length, reproductive capacity, and spatiotemporal distribution of blue sharks based on biological data and capture location information collected in the western Indian Ocean from 2010 to 2020. The objective of this study is to provide reliable biological information important in performing future stock assessments vital for species conservation in this region. A total of 791 male (33–249.5 cm FL) and 803 female (12–349.6 cm FL) blue sharks were collected in the West Indian Ocean. We used the morphology of the sexual organs to ascertain their sexual maturity. Results show that the observed size at 50% sexual maturity of male blue sharks in the West Indian Ocean was 161.4cm FL (192.4 cm TL) for males and 179.3 cm FL (213.9 cm TL) for females based on logistic curve analysis. The average litter size of pregnant blue sharks was 33.7 pups. There were significant differences in the distribution of blue shark individuals with different sexual maturity levels in different quarters (*p* < 0.05). This study suggests that the area near the equator in the Indian Ocean from October to March of the following year may be the mating ground for blue sharks, while the temperate waters in the Indian Ocean are the nursery ground and parturition ground for pregnant and juvenile throughout the whole year. Therefore, it is recommended to adopt a more scientific and reasonable operational method in these areas.

## 1. Introduction

The blue shark (*Prionace glauca*) belongs to the family *Carcharhinidae* of the order *Carcharhiniformes*. As viviparous fish, blue sharks have lifespans and reproductive patterns similar to those of mammals [1], positioning them as one of the most fecund shark species in terms of reproductive potential. As a highly migratory species, the blue shark is widely distributed in the upper layers of the world's prominent tropical and temperate oceans. The preferred temperature for this species falls within the range of 12–25 °C. The distribution and migration of blue sharks are influenced by water temperature, reproductive conditions, and food abundance, and are subject to large seasonal variations with significant seasonal variation [2]. Blue sharks also commonly show vertical migration patterns [3,4,5]. These species are top bycatch species due to their constant presence in the same water column as primary targets, such as large species like tunas and swordfish. As a result, their populations are on the decline and they are Near Threatened according to the IUCN red list for threatened species [6].

Since the 1990s, a continuous decline in the catch per unit effort (CPUE) of blue sharks has been observed across all major oceans [7]. The western Indian Ocean is an important fishing ground for longline tuna fisheries [7], and as the main bycatch species of longline tuna fishing vessels, the distribution of blue sharks overlaps extensively with that of tuna and swordfish. Consequently, the status of the blue shark stock has persistently suffered from the effects of tuna fishing vessels. Large pelagic sharks account for more than 50% of the chondrichthys bycatch in pelagic longline fisheries, with blue sharks accounting for the largest catches in temperate and sub-tropical areas [8]. According to observers’ data records spanning the period from January 2006 to December 2018, blue sharks represented 60% of the bycatch in the commercial longline tuna fishery in Indonesia [1]. According to data from the IOTC (Indian Ocean Tuna Commission) reports between 2015 and 2019, the annual landing of blue sharks averaged 26,691 tons [9,10]. Due to limited data sources, our understanding of shark bycatch is limited, and, as a result, our knowledge of shark populations is limited. 

The reproductive biology of the blue shark in the Atlantic and Pacific oceans has been extensively studied [11,12]. However, research on this topic in the Indian Ocean is relatively scarce [12], with only a few studies in limited areas [1,10,13,14,15]. Jolly analyzed 205 blue shark samples caught by fishery vessels along the southeastern coast of South Africa and found that the length at sexual maturity was 201.4 cm TL for males and 194.4 cm TL for females [16]. They also collected one pregnant female and 55 postpartum females and found that males were more commonly seen than females in the southern waters of South Africa. Other researchers [10] estimated the length at first sexual maturity at 201.7 cm SFL for males and 142.0 cm SFL for females based on the analysis of 266 blue shark samples from the southwestern Indian Ocean and found that the length at sexual maturity for females was consistent with the parameters used in the current IOTC assessment model. In other locations, researchers reported differences in blue sharks’ length at sexual maturity and fecundity [17,18,19].

The present study aimed to enhance our understanding of blue sharks’ reproductive biology in the Indian Ocean and to provide a basis for effectively managing and conserving this vital resource. Specifically, with the research on key reproductive indicators including length distribution, sex ratios, pregnancy rates, litter size, sexual maturity, and spatiotemporal distribution of blue sharks, this study provided basic biological data for the assessment of blue shark stock and the conservation and management of blue shark resources in the Indian Ocean. Due to limitations in manpower and resources, we were only able to collect biological samples of blue sharks in the western Indian Ocean for analysis, providing only partial details for the study of blue shark reproductive biology in the Indian Ocean. 

## 2. Materials and Methods

### 2.1. Samples and Data Collection

**Figure 1:**
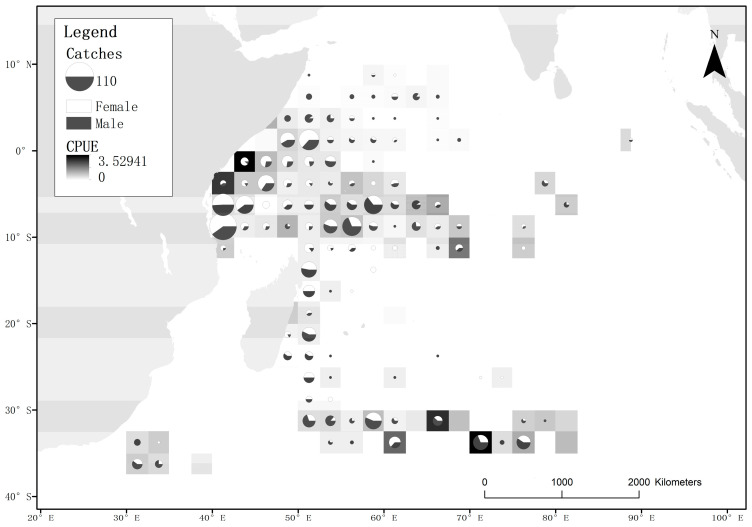
Distribution of blue shark samples and sex ratio in the area of study during the study period. The size of the circles represents the number of captured samples in that area. The ratio of black and white areas in the circle indicates the proportion of male and female samples, with white representing females and black representing males. The shading of the squares indicates the magnitude of the nominal CPUE.

The data used in this study were collected by observers from the Chinese distant-water fishing scientific observation project aboard longline vessels targeting Indian Ocean tuna. The observers received training per the requirements of IOTC for data collection. The recorded data include operation location, number of hooks, hook time, bait usage, weather conditions, sea surface temperature and individual biological information of the caught specimens (species name, fork length, sex, maturity status, male clasper length, female oviduct gland width, liver weight and sex ratio of embryos in pregnant females). The data used in this study were obtained from observers onboard Chinese distant-water tuna longline vessels from July 2010 to June 2020 in the western Indian Ocean, covering the geographical range, from 10° N to 39° S and from 23° E to 90° E (Figure 1). The observers recorded 2674 sets and 8,267,659 hooks, capturing a total of 1594 blue sharks (Table 1). The captured location, fork length, sex, maturity stage, and catch number data of blue sharks were analyzed and organized using Excel and visualized using ArcGIS and the R software.

To some extent, the CPUE reflects the abundance of the blue shark population in a research area. The higher the CPUE value, the more blue sharks there are in that area. The CPUE of the blue shark catch location data was divided into 2.5° × 2.5° grids. The CPUE of blue sharks was calculated and plotted using the following formula based on data from catches, distribution, hook numbers, and situations:

CPUE = C/E

where C is the total catch in a certain sea area, and E is the total number of hooks in that sea area (thousands).

### 2.2. Length Data

With a grouping interval of 20 cm, a frequency distribution graph of fork length for male and female blue sharks was plotted. The two-sample Kolmogorov-Smirnov (K-S) test for goodness of fit was used to examine whether there were significant differences in the fork length distribution between male and female blue shark samples. The *t*-test (two-tailed independent samples *t*-test) was used to examine whether the mean fork length of male and female samples followed a normal distribution and to see whether there were significant differences in the fork length distribution between male and female blue shark samples.

### 2.3. Length at Sexual Maturity

The sex and sexual maturity of the blue sharks were determined by dissecting their reproductive systems. For male blue sharks, sexual maturity was divided into three stages [20,21]: 1. Juvenile, with uncalcified claspers and thin testes, and semen not present; 2. Adolescents, with partial calcification of the claspers, thickening of the testes, and semen may be present in the testes; 3. Adult, with fully calcified and stiff claspers, enlarged and predominant testes, and semen may be present. Juvenile and adolescent indicate immaturity, while adult indicates sexual maturity. 

For female blue sharks, sexual maturity was divided into five stages [20,22]: 1. Juvenile, with a thin and white uterus and very small ovary; 2. Adolescent, with a thin and white uterus but partly enlarged posteriorly, and ovary developing but no developing follicles; 3. Adult, with enlarged and empty uterus and developing follicles; 4. Pregnant, with an enlarged uterus containing embryos or fertilized eggs; and 5. Postpartum, with a greatly enlarged, flaccid uterus, and a distended placenta or umbilical cord may be present in the uterus. The juvenile and adolescent indicate immaturity, while the adult, pregnant, and postpartum stages indicate sexual maturity.

The maturity stage of each sample was converted to a binomial distribution value (immature = 0, mature = 1) for statistical analysis. The ratio of mature individuals in each clasper length group was calculated for both male and female blue sharks using 20 cm intervals. A logistic model was used to fit the relationship between the mean values of each clasper length group and the ratio of sexually mature individuals, and the length at which 50% of blue sharks reached sexual maturity was calculated [23].

*P* = 1/(1 + *e*^−(−*α*×*FL*−*β*)^)

In the equation above, *FL* represents the mean value of each length group, *P* is the proportion of mature individuals in each interval, and *α*, *β* are the coefficients of the logistic curve. 

### 2.4. Reproductive Capacity and Liver Weight

The liver is an organ that stores, absorbs and transports nutrients in fish, and the liver index varies according to gender and developmental stage. At the beginning of the pregnancy, relatively little energy is required for the development of fertilized eggs, but a large amount of energy is required for embryo development during the larval stage, which mainly comes from the liver [18,24]. In this study, the viewpoint will be validated by recording the liver weight, fork length, and sexual maturity of the blue sharks. The number of fetuses in pregnant females will be recorded to analyze whether the number of offspring is related to length, and to further infer the reproductive pattern of females.

## 3. Results

### 3.1. Analysis of Fishing Information

**Figure 2:**
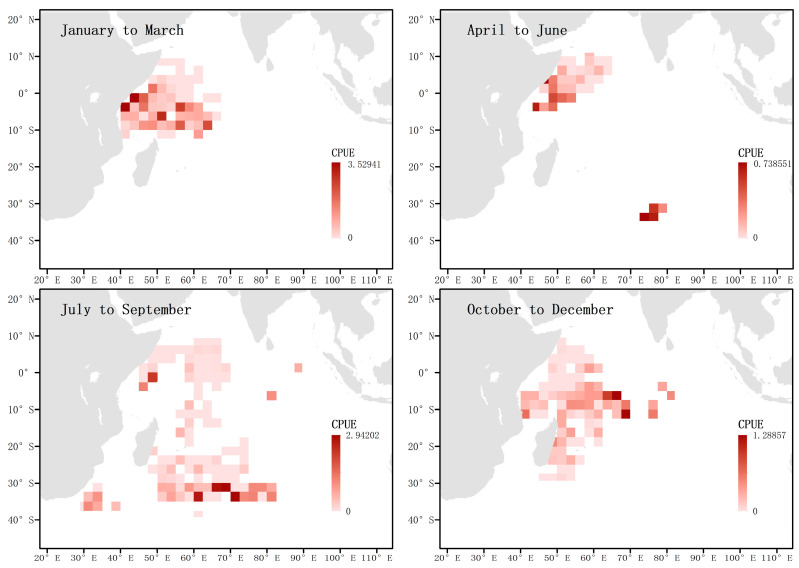
Investigation of quarterly CPUE of the blue shark in the surveyed sea areas. The shading of the squares indicates the magnitude of the nominal CPUE.

Higher CPUE values in certain areas reflect a higher density of blue sharks during that period. By investigating the CPUE of blue sharks in the western Indian Ocean during each season, the results showed (Figure 2) that the CPUE was relatively high along the Tanzanian coast in the first and second quarters, in the temperate waters of the Southern Indian Ocean in the third quarter, and in tropical waters in the fourth quarter.

### 3.2. Length Distribution

**Figure 3:**
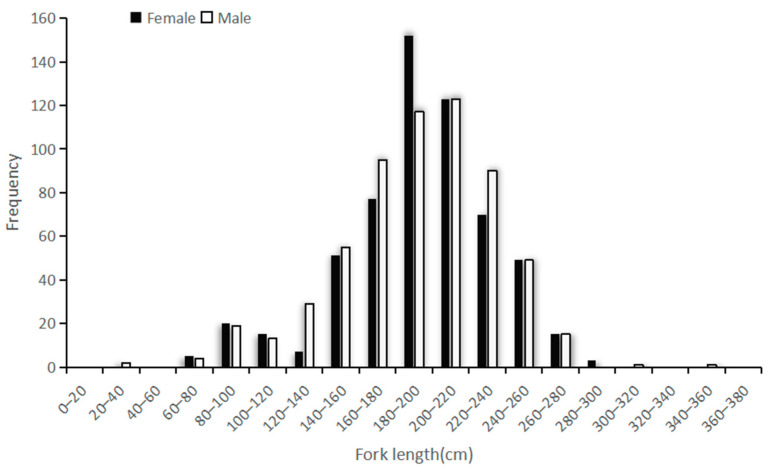
Frequency distribution of fork length (cm) for male and female blue sharks. White squares represent the number of female samples, and black squares represent the number of male samples.

This study collected a total of 1594 blue sharks, including 813 male samples, accounting for 51%, with an average fork length of 192.8 cm, and 781 female samples, accounting for 49%, with an average fork length of 194.3 cm. The length frequency distribution analysis of male and female samples is indicated in Figure 3; the fork length of male and female blue sharks followed a normal distribution (K-S test *p* = < 0.05), and no significant difference in the distribution between the two samples (D = 0.048 < 0.05) was observed. In the 180–200 cm FL group, the number of females is significantly higher than that of males, while in the 120–140 cm FL, 160–180 cm FL, and 200–240 cm FL groups, the number of males and females is almost the same. The group with the highest number of male samples is 200–220 cm FL, accounting for 26%; the group with the highest number of female samples is 180–200 cm FL, accounting for 20%.

### 3.3. Length at Sex Maturity

**Figure 4:**
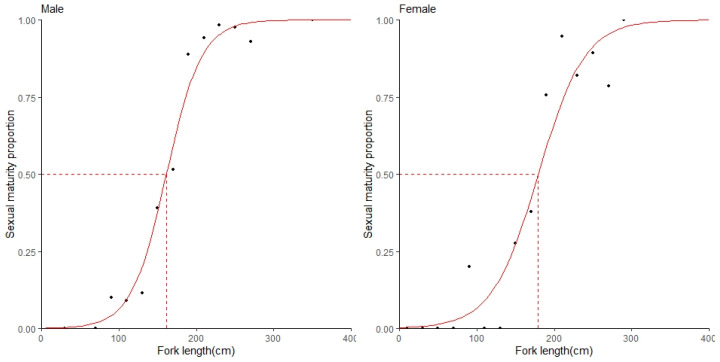
The relationship between sexual maturity proportion and fork length (cm) in male and female blue sharks. The red curve represents the logistic curve.

The pattern of changes in the sexual maturity rate of male and female blue sharks with body length can be well-fitted using a logistic model (Figure 4). The logistic equation for the median sexual maturity rate in each length group of male blue sharks:

*P* = 1/(1 + *e*^(−0.044×(L^_F_^+7.1069))^)

The parameters are *α* = 0.044 and *β* = −7.1069.

The logistic equation for the sex maturity rate of female blue sharks in relation to the median fork length of each length group:

*P* = 1/(1 + *e*^(−0.033×(L^_F_^+5.990))^)

The parameters are *α* = 0.033 and *β* = −5.990. When *P* is equal to 0.5, we can take this into the equation and conclude that the sizes at 50% maturity of males and females were 161.4 cm fork length (FL) and 179.3 cm FL, respectively. Within the body length range of 200–240 cm FL, there was still a certain proportion of females that had not reached sexual maturity, while most males reached sexual maturity in this length range. The length at which females reached sexual maturity was greater than that of males.

**Figure 5:**
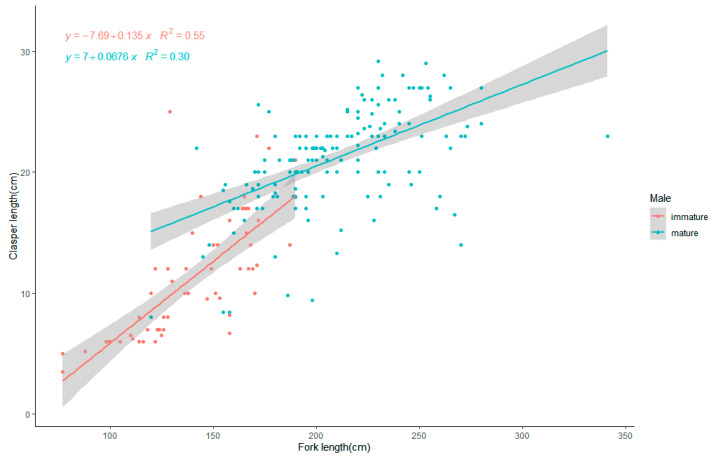
The relationship between the fork length (cm) and the clasper length (cm) of the blue sharks. The blue dots represent maturing male individuals, and the blue line represents the linear regression line for these individuals. The red dots represent matured male individuals, and the red line represents the linear regression line for these individuals. The shaded area represents the 95% confidence interval.

Analysis of the relationship between fork length and clasper length in male blue sharks (Figure 5) shows that the range of fork lengths for sexually mature males was 100–341 cm, and the range of clasper lengths for matured blue sharks was 8.0–29.2 cm. Clasper length in males shows a clear positive correlation with fork length, with immature males developing more quickly, resulting in a steeper regression line between clasper length and fork length. In mature males, gonad development slows down, resulting in a lower slope of the regression line. The relationship between fork length and clasper length in mature males is expressed as CL = 0.068 FL + 7.0, while the relationship in immature males is expressed as CL = 0.135 FL − 7.69. Here, CL represents clasper length, and FL represents fork length.

### 3.4. Reproductive Capacity and Liver Weight

**Figure 6:**
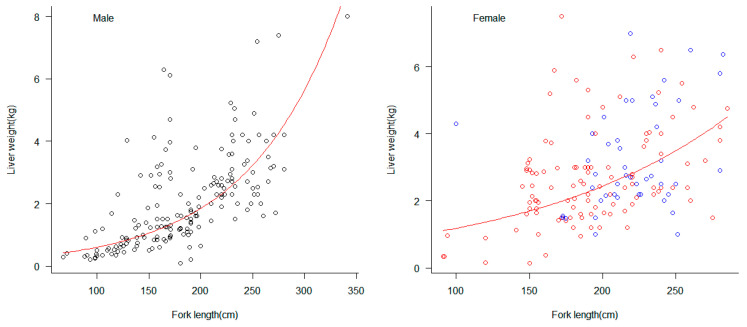
The exponential relationship between liver weight (kg) and fork length (cm) in male and female blue sharks. The circles with black borders represent male individuals, the circles with red borders represent non-pregnant female individuals, and the circles with blue borders represent pregnant female individuals. The red curve represents the fitted exponential model. The fitting relationship between fork length and liver weight was LW = 0.198*e*^0.0112FL^ for males and LW = 0.574*e*^0.007FL^ for females. LW represents liver weight, FL represents fork length.

The observed range of liver weights for male blue sharks was 0.10–8.00 kg, with an average weight of 2.05 kg. For female samples, the range of liver weights for non-pregnant individuals was 0.15–7.50 kg, with an average weight of 2.69 kg. The range of liver weights for pregnant females was 1.01 kg–7.13 kg, with an average weight of 3.43 kg. *t*-test analysis showed that the average liver weight of female blue sharks was larger than that of males. The exponential model was used to fit the relationship between body length and liver weight for male and female blue sharks, and the results showed (Figure 6) that the liver weight-fork length relationship for male individuals had a higher fit to the exponential model than that for females.

**Figure 7:**
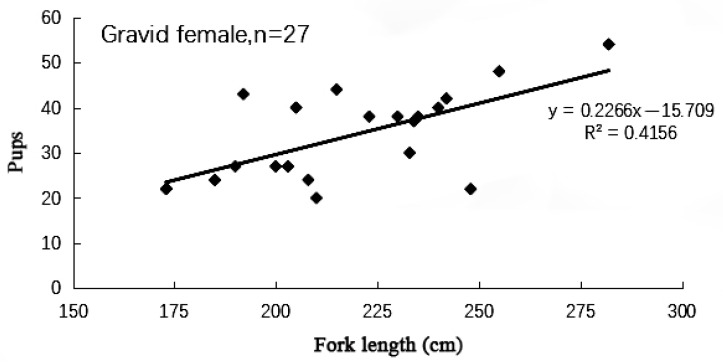
The relationship between maternal fork length (cm) and the number of pups of blue sharks.

The range of length at sexual maturity of female samples of the blue shark was 100–287 cm. The width of the oviducal gland of female specimens changed with the reproductive cycle. It increased during ovulation and gestation periods, and 95% of sexual maturity females have an oviducal gland width greater than 2.5 cm. The number of embryos carried by pregnant blue sharks ranged from 20 to 54 (Figure 7), with an average of 33.7. Records show that the number and total length of male embryos are slightly greater than those of female embryos. Pearson correlation analysis shows that the number of embryos carried by blue sharks is positively correlated with the fork length of female blue sharks (*p* = 0.002 < 0.05, Multiple R = 0.645).

### 3.5. Sex Ratio

**Figure 8:**
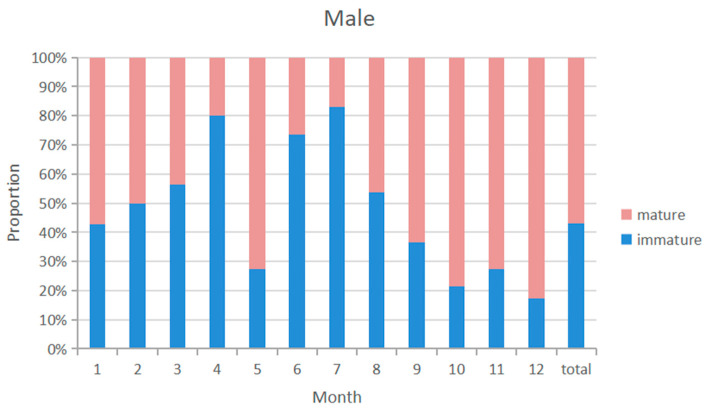
Sexual maturity proportion of male samples by month from 2010 to 2020.

**Figure 9:**
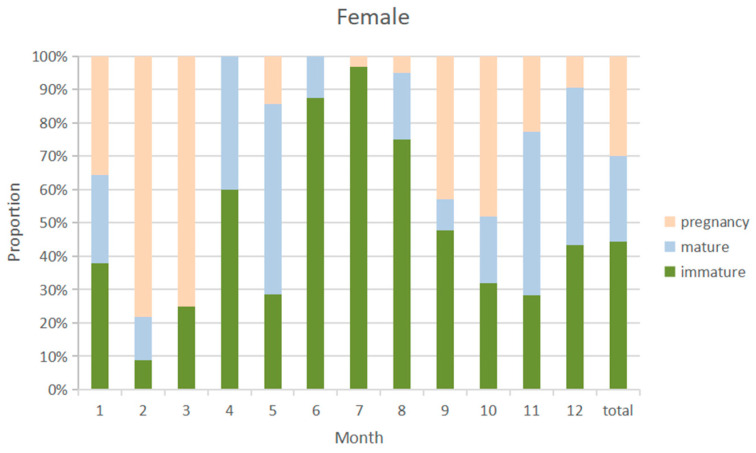
Sexual maturity proportion of female samples by month from 2010 to 2020.

The overall sex ratio of blue shark samples was close to 1:1. The monthly distribution of the sexual maturity ratio of male and female blue shark samples was similar (Figure 8 and Figure 9), with a higher probability of catching immature individuals from April to August and a higher probability of catching sexual maturity individuals from January to March and from September to December. The highest proportion of immature individuals was caught in July. The highest proportion of pregnant females was caught from February to March, with immature female individuals accounting for 44.3% of the samples and mature individuals accounting for 53.4%, while pregnant individuals accounted for 29.8%.

### 3.6. Spatiotemporal Distribution of Sexual Maturity

This study divided the overall samples of the blue sharks into four groups for comparative analysis [25]: juvenile individuals below 125 cm FL, immature individuals above 125 cm FL, sexually mature females and males, and pregnant females.

**Figure 10:**
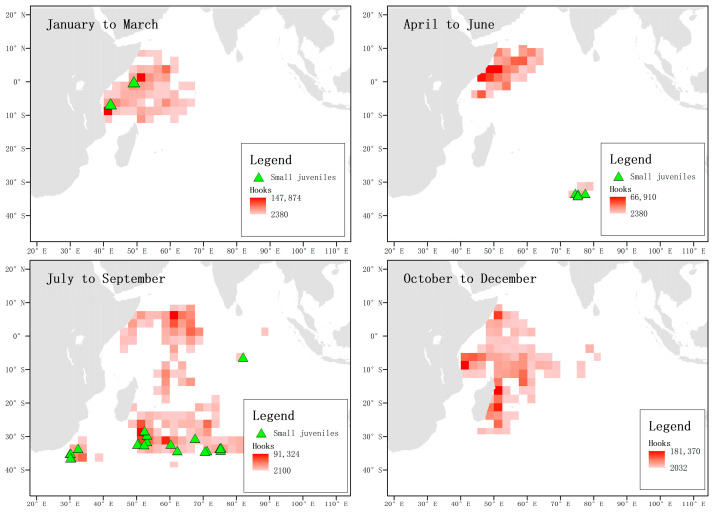
Location and hooking information of juvenile blue sharks below 125 cm FL. The green triangles represent the locations where matured and non-pregnant female individuals were captured, and the shading of the squares indicates the number of hooks.

When analyzing the location and hooking situation of juvenile blue sharks below 125 cm FL captured in each season, it was found that between April and September, the frequency of occurrence was higher in the temperate waters between 25° S and 40° S in the western Indian Ocean. However, in the other quarters, juvenile blue sharks below 125 cm FL were not captured in large numbers in the waters north above 25° S (Figure 10).

**Figure 11:**
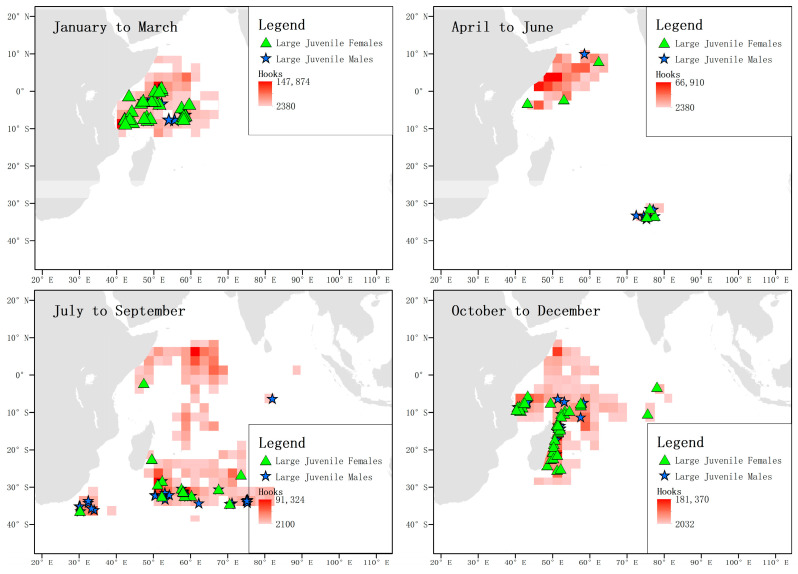
Distribution of male and female immature sharks above 125 cm FL. The green triangles represent the locations where immature female individuals were captured, and the blue stars represent the locations where immature male individuals were captured. The shading of the squares indicates the number of hooks.

By analyzing the distribution of immature individuals of both sexes greater than 125 cm FL in each season, it was found that the distribution pattern was the same throughout the year. In the first quarter, they were mainly concentrated in the waters near the equator, then left the area between April and June, and appeared more frequently in the temperate waters between July and September. Between October and December, they were mainly concentrated along the coast of Tanzania and the eastern coast of Madagascar (Figure 11).

**Figure 12:**
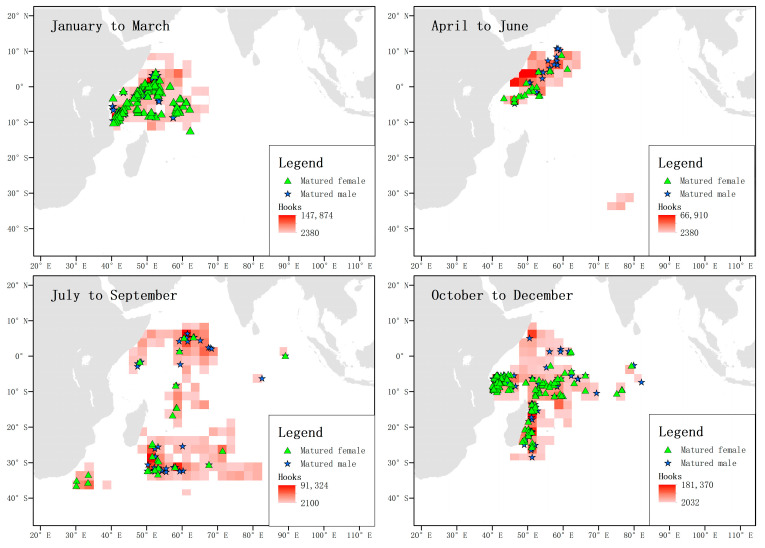
Locations and total catch of matured blue sharks. The green triangles represent the locations where matured and non-pregnant female individuals were captured, and the blue stars represent the locations where matured male individuals were captured. The shading of the squares indicates the number of hooks.

When analyzing the location and total number of hooks of captured matured females and males in each season, it was found that the activity ranges of matured males and females highly overlapped from October to March of the following year, but not in the second and third quarters. From July to September, matured females migrated southward to temperate waters, while their numbers near the equator were relatively low, during which time the number of males near the equator predominated. From October to December, sexually mature female individuals were concentrated in waters south of the equator. It is worth noting that between July and September, a certain number of matured females appeared in the southwest coastal waters of South Africa, while matured males did not appear in this area but were distributed in offshore waters (Figure 12).

**Figure 13:**
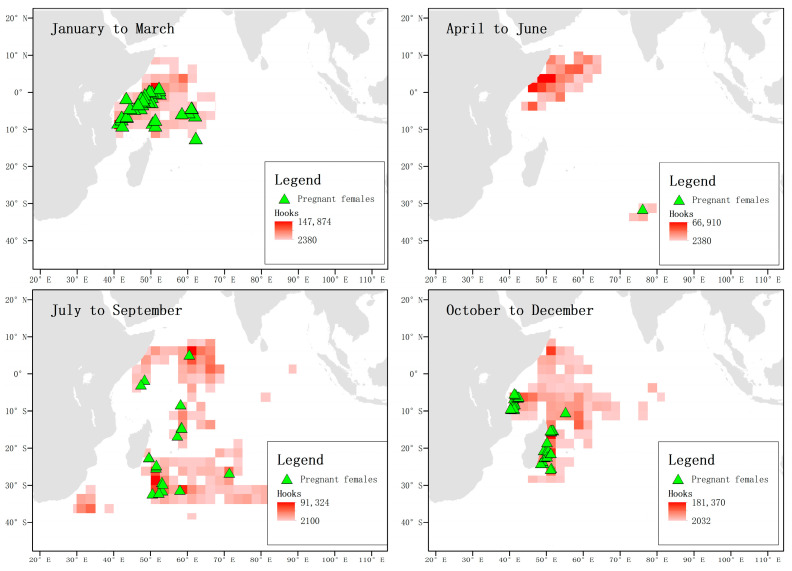
Distribution and total catch of pregnant blue sharks. The green triangles represent the locations where pregnant female individuals were captured, and the shading of the squares indicates the number of hooks.

When analyzing the captured pregnant female blue sharks in each season and the total number of hooks, it was found that between January and March, pregnant females were mainly distributed in the waters near the equator, while no pregnant females were captured in this area between April and June. From October to December, the frequency of pregnant blue sharks near the equator was low, while their frequency was high in the waters near 30° S in the temperate zone. From October to December, pregnant blue sharks were mainly distributed in the coastal waters of Tanzania and the east coast of Madagascar, and no pregnant females were found in the waters near the equator (Figure 13).

## 4. Discussion

### 4.1. Sexual Maturity Length

At present, the method of determining the sexual maturity of male and female blue sharks through the development of their reproductive organs has been widely applied. Previous studies mainly relied on measuring the length and calcification level of the claspers, the development of testes, and the presence of sperm in the testes to determine the sexual maturity of male individuals; while the development of the uterus and ovaries were used to determine the sexual maturity of female individuals [10,16,18,20,23,26]. Other methods include measuring the width of the female oviducal gland [26], calculating the gonadosomatic index [24], and observing bite marks on the body [18]. Although the use of bite marks as a means of determining female sexual maturity has been suggested, it is not a reliable or advisable method of differentiation, as stated by Pratt [18].

This study converted the preliminary 50% sexual maturity fork length for males (161.4 cm FL) and females (179.3 cm FL) of the blue shark into 50% sexual maturity total length (192.4 cmTL for males and 213.9 cmTL for females) using the relationship equation between fork length and total length (FL = 1.73872 + 0.82995 TL) [18]. The results of this study were significantly lower than the average values (212 cm for males) reported in other studies for blue sharks [27]. Additionally, the results of this study were slightly lower than the 2021 IOTC report on the status of blue sharks in the Indian Ocean, which showed a male sexual maturity length of 201 cmTL. Similar results were also reported in studies conducted by [10,28]. The length at sexual maturity for females was similar to the global average reported size [27] but higher than the 194 cmTL reported in the 2015 IOTC report on the status of blue sharks in the Indian Ocean and the study by [10]. The large differences in the results of various studies may be due to differences in areas where samples were collected and environmental conditions, as well as the use of different body length measurement methods (Table 2) [17,18,19]. Therefore, when selecting evaluation model parameters, it is recommended to comprehensively consider the research data from the entire Indian Ocean to obtain more reliable results.

### 4.2. Reproductive Capacity

This study obtained an average litter size of 33.7 pups. As of now, data on the reproductive capacity of the blue shark is only available for the Atlantic and Pacific oceans, with an average litter size of 30 pups [27]. 

This study found that the relationship between male length and liver weight fits an exponential model, while the relationship between female length and liver weight does not fit the exponential model well. Typically, the energy required for embryonic development during early pregnancy is relatively low, while a significant amount of energy is needed for embryonic development during the juvenile stage, primarily derived from the liver [18,24]. Additionally, the liver provides energy for gonad development in the blue shark, and liver weight in matured individuals is significantly greater than in immature individuals [18]. The placenta of a blue shark mother extracts nutrients from the liver to support fetal growth and development, so the liver weight of pregnant females is significantly lighter than that of non-pregnant females [23]. This is the main reason the relationship between female liver weight and body length cannot be well fitted by an exponential model [29,30,31,32].

### 4.3. Migration Patterns

**Figure 14:**
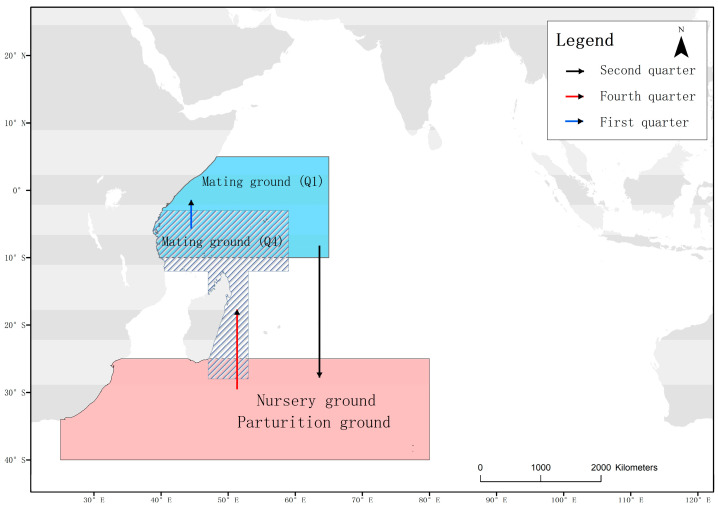
Migration routes of the blue shark, with arrows indicating the direction of movement for pregnant and sexually mature females. The blue area represents the potential mating area for the first quarter (Q1), the gray diagonal area represents the potential mating area for the fourth quarter (Q4), and the pink area represents the nursery and parturition areas. Arrows indicate migration directions.

This study suggests that juvenile blue sharks below 125 cm FL are usually located in temperate waters between 35° S and 40° S. Pregnant matured females, and immature juveniles over 125 cm FL have the same distribution pattern in each season, concentrating near the equator between January and March, migrating south to temperate waters between April and September, and migrating north to coastal waters of Tanzania and Madagascar from October to December. Earlier research has indicated that while blue sharks residing in the Indian Ocean exhibit variation in their body sizes and sex ratios, they still fall under the same stock and do not experience seasonal reproductive isolation [33,34]. Large individuals are mainly found in tropical regions, while smaller individuals are predominant in temperate regions [35,36,37]. Stock abundance often increases with distance from land [14,38]. However, Jolly [16] reported sex segregation among blue sharks in the southern waters of South Africa, where male samples were more common than female samples, indicating an uneven distribution of blue sharks at different maturity levels. Based on these observations, a preliminary migration map of blue sharks in the western Indian Ocean can be established (Figure 14). This migratory pattern is similar to Nakano’s findings in the Pacific Ocean [23] but differs significantly from the distribution of blue sharks in the North Atlantic [5,15,39,40].

The migration of blue sharks is mainly accomplished through ocean current systems [19,41]. Tagging studies have shown that blue sharks in the Pacific and Atlantic Oceans undertake large-scale migrations [14,42,43]. Survey studies conducted in the equatorial waters of the Indian Ocean between 12° N and 10° S found that pregnant female blue sharks were mainly concentrated in the waters between the east coast of Africa and 55° E, 2° N–6° S [35]. It was also found that blue shark juveniles and sub-adults mainly concentrate in the temperate waters of the southwest Indian Ocean and the southeast Indian Ocean, which are considered the two main breeding areas of blue sharks in the Indian Ocean. The breeding areas in the Indian Ocean are distributed in temperate waters, especially in the southwest and southeast Indian Ocean near South Africa and Australia [35]. This is consistent with the results of the present study, where small blue sharks accounted for over 80% of the catches in this area.

Carrera-Fernández [26] found that the captured samples of blue sharks in the nearshore waters of New Zealand and western Mexico were mostly medium-sized and small individuals, and they believed that blue sharks in these areas were heavily impacted by fishing. Based on previous studies [18,23] and the results of this study, it is believed that the reason why the collected blue shark samples in the New Zealand waters were mostly medium and small individuals is that this area serves as a nursery and breeding ground for blue sharks, while in western Mexico, overfishing of blue sharks may have led to the absence of large individuals.

### 4.4. Reproductive Season

This study observed that the distribution of matured male and female blue sharks overlapped highly in the first and fourth quarters, and was concentrated in certain areas. There may be two mating areas for blue sharks in the western Indian Ocean. The first being near the equator in the first quarter, and the second in the coastal waters of Tanzania and eastern Madagascar in the second quarter. Pregnant blue sharks captured in the western Indian Ocean are most abundant from September to March of the following year, with the highest proportion from January to March. Although the total number of recorded catches was lower from January to March, more pregnant blue sharks were captured during this period. Pregnant blue sharks mainly concentrated in the area between 40° E and 60° E and between 5° N and 10° S in the first quarter. The frequency of pregnant individuals in the sample was lowest from December to January (Figure 9). Pregnant females leave the area near the equator and migrate south in February and March. Juvenile blue sharks below 125 cm FL have limited swimming abilities and inhabit temperate waters throughout the year, indicating that pregnant females may give birth in the second or third quarter. This study demonstrates that the breeding sites of blue sharks exhibit significant seasonal differences.

This study found that the proportion of immature individuals was higher in the temperate waters of the southern Indian Ocean, indicating that the activity range of male and juvenile blue sharks is usually closer to the southern latitudes, which is consistent with the results of studies by Coelho and Vandeperre [5,35]. This further suggests that temperate waters between 35° S and 40° S in the southern Indian Ocean may be a potential area for blue shark reproduction and juvenile growth. Females that mate and become pregnant near the equator in the first quarter may migrate southwards and give birth in the temperate waters in the third quarter, while females that mate and become pregnant in the offshore waters of Tanzania and the eastern coast of Madagascar in the fourth quarter may migrate southwards and give birth in temperate waters in the second quarter of the following year. The distribution of blue sharks can be further inferred by studying the changes in their prey organisms [44].

Many studies have suggested that blue sharks have a seasonal reproduction scheme, with an embryo development period of 9–12 months, and pregnant females usually give birth in spring or summer [18,19,20,23]. Females in the northwest Atlantic reach sexual maturity at 5 years old and begin mating in May or June of spring, with the offspring being born from April to July of the following year [17]. Amorim [45] suggests that the mating of blue sharks in the southwest Atlantic occurs from December to February of the following year, while Hazin [38] suggests that the mating of blue sharks in northeast Brazil occurs from March to May. Researchers have shown that the time and area of ovulation and fertilization of blue sharks in the western Indian Ocean equatorial region is similar to that of the southwestern Atlantic [14].

Currently, little is known about the bycatch of sharks, and the data sources for assessment models are limited, resulting in a limited understanding of the population status of sharks. IOTC uses a stock synthesis (SS) model to assess the status of blue shark resources in the Indian Ocean [9]. Age, growth, and reproductive parameters obtained from reproductive biology studies are key to improving the accuracy of population assessments for these species [10,12]. All of the blue shark samples in this study were obtained from commercial fishing, so the distribution pattern of the samples may be closely related to fishing activities and may deviate from the actual distribution. Because tuna longline fishing operations undergo seasonal changes, we can only observe changes in the concentration of blue sharks in various marine areas as the longline fishing area changes, but this does not affect our observations of species richness and distribution of blue sharks in a specific marine area at a particular time. It is difficult to obtain the weight and age data of blue sharks, and collecting the reproductive biology data of blue sharks using the original method will lead to their deaths. Observers may face the risk of being bitten while measuring. Therefore, we need more advanced and convenient methods, such as ultrasonic detectors, to ensure that the sharks caught simultaneously are released alive while reducing the risk of personal injury. This represents a significant increase in costs, and we hope to find better solutions in future research. The length of 50% sexual maturity of blue sharks in this study area is similar to that in other areas, but without previous research in this area, we cannot assess whether this species is overfished. If subsequent research finds that 50% of sexual maturity length has decreased, this means that it has been overfished.

## 5. Conclusions

This study observed that the average litter size of pregnant blue sharks was 33.7 pups, and its fecundity was significantly higher than that of other pelagic sharks, which may be the reason why its population still did not decline significantly under the influence of bycatch. This study suggests that the area near the equator of the Indian Ocean from October to March of next year may be the mating area of blue sharks, while the temperate waters of the Indian Ocean are the area where blue sharks spawn and grow. Therefore, it is suggested that more scientific and reasonable management methods should be adopted when fishing activities are carried out in these waters to reduce the bycatch of blue sharks and ensure the survival of the released sharks.

## Figures and Tables

**Table 1:** Observation trips data of longline fishing vessels in the western Indian Ocean during 2010–2020.

Start Time	End Time	Vessels	Longitude Range	Latitude Range	Operations	Samples
July 2010	January 2013	2	29.1–62.35	−12.29–2.5	111	148
October 2013	September 2014	3	47.16–89.54	−33.2–0.19	190	253
December 2015	April 2017	5	23.51–82.1	−27.52–4.16	548	539
April 2017	January 2018	4	25.9–82.1	−34.9–10.9	532	380
May 2018	January 2019	3	45.4–71.9	−27.15–10.9	330	121
April 2019	June 2020	4	45.13–72.5	−30.4–9.9	707	149

**Table 2:** Comparison of sexual maturity of each study.

Source	50% Maturity Size Male (cm)	50% Maturity Size Female (cm)	Simples	Area of Research
Jolly 2013	201.4 (TL)	194.4 (TL)	205	West Indian Ocean
Murua 2021	201.7 (SFL)	142.0 (SFL)	226	West Indian Ocean
Vargehese 2016	207.11 (TL)		26	eastern Arabian Sea
IOTC 2015	201 (TL)	194 (TL)		
World average	212	208		
This study	161.4 (FL)	179.3 (FL)	1594	West Indian Ocean
	179.3 (FL)	213.9 (TL)		

## Data Availability

Data is available upon request from the corresponding author.
